# Children are not little adults: blood transfusion in children with burn injury

**DOI:** 10.1186/s41038-017-0090-z

**Published:** 2017-08-15

**Authors:** Tina L. Palmieri

**Affiliations:** Shriners Hospital for Children Northern California and the University of California, Davis, 2425 Stockton Blvd, Suite 718, Sacramento, CA 95817 USA

**Keywords:** Blood transfusion, Pediatric, Burn injury

## Abstract

Blood transfusion in burns larger than 20% total body surface area (TBSA) are frequent due to operative procedures, blood sampling, and physiologic response to burn injury. Optimizing the use of blood transfusions requires an understanding of the physiology of burn injury, the risks and benefits of blood transfusion, and the indications for transfusion. Age also plays a role in determining blood transfusion requirements. Children in particular have a different physiology than adults, which needs to be considered prior to transfusing blood and blood products. This article describes the physiologic differences between children and adults in general and after burn injury and describes how these differences impact blood transfusion practices in children.

## Background

Children and adults have different physiologic and hematologic systems, which impacts therapeutic interventions and their efficacy. In addition, children of different ages have different physiology and anatomy, which further complicates treatment. For example, an infant has a higher metabolic rate than an 8-year-old, a larger body surface area to mass ratio, and a markedly smaller blood volume. Hence, different strategies need to be employed when treating children of different ages. These differences are accentuated in burn injury, which further alters metabolism, anatomy, and physiology. Understanding the differences among children of different age groups is essential to optimize the use of blood transfusion in children. This article will discuss how differences in the physiologic, hematologic, metabolic, and immunologic systems in burned children impact blood transfusion requirements. Although this article describes how children differ from adults in terms of factors with impact on blood transfusion, the unique primary aim of this article is to understand how burned children are impacted by blood transfusion and describe optimal transfusion practices in burned children (Table 1).

**Table 1:** Summary of transfusion considerations in burned children

1. Despite a smaller stature, burned children have a greater body surface area per mass than adults.
2. Cardiac function, mean blood volume, and normal hemoglobin levels are age-dependent in children; hence, children have a higher blood transfusion/unit volume ratio.
3. The optimal hemoglobin threshold for initiating a blood transfusion in burned children has not yet been defined.
4. Hyperkalemia associated with blood transfusion poses a significant risk in children, and potassium levels should be monitored in children receiving >20 ml/kg transfusion volume.
5. The maximal allowable blood loss (MABL), i.e., the volume of blood that can be lost in an operation without transfusing blood, can be calculated from the following (Hct = hematocrit, EBV = estimated blood volume): MABL = [(Hct_start_ − Hct_target_)/Hct_start_] × EBV.

## Review

### Children and adults have differences in hematologic and physiologic characteristics

Children clearly have a smaller stature than adults, yet their requirements may actually exceed those for adults on a kilo per kilo basis. For example, young children have a greater body surface area per mass than an adult, and the distribution of that mass is different than in adults. This impacts burn size determination, intravenous fluid requirements, and blood transfusion requirements.

Even the most essential body systems are impacted by the differences between children and adults. Heart rate measurement is simple, yet there are important differences between children and adults that should be considered when instituting burn treatment. The baseline heart rate in a child is higher than that in an adult and varies with age [1]. Burned children have a higher cardiac output and heart rate than unburned children, which can predispose them to heart failure.

Cardiac function also differs with age. As a baseline, a newborn child’s myocardium is at near maximum function; hence, the newborn may not be able to compensate for decreased oxygen carrying capacity by increasing cardiac output after injury [2]. In other words, an infant increases heart rate rather than contractility to increase cardiac output. In the burned child, whose hypermetabolic rate adds further demand to an already stressed system, tachycardia is increased. Hence, burned infants are at particular risk for heart failure after injury. Beta blockade will be problematic, as lowering the heart rate will also lower cardiac output. Finally, myocardial ischemia could occur due to decreased oxygen delivery capacity in the newborn or very young infant, which may in part contribute to the increased mortality of burned children less than 2 years.

A second difference between adults and children is in blood volume. A child’s mean blood volume approximates 70 ml/kg, which exceeds adult blood volume/weight calculation. This increased blood volume/unit mass impacts a variety of body functions. As indicated above, oxygen consumption in children is higher; in addition, cardiac output to blood volume ratio is also higher in children than in adults [3, 4].

Normal hemoglobin levels in children are age-dependent and also differ from adults. Children are born with a hemoglobin level approximating 19 g/dL and have a nadir of 11.2 g/dL at approximately 2–3 months of age. Eventually, a child’s hemoglobin stabilizes at approximately 13 g/dL [5]. In infants, fetal hemoglobin can play a role in oxygen delivery, thus decreasing the efficacy of the at-birth oxygen delivery. At birth, fetal hemoglobin constitutes 70% of the child’s hemoglobin. At 6 months of age, however, only a trace of fetal hemoglobin remains [6, 7]. In fetal hemoglobin, red blood cell life span is decreased by 30 days (from 120 to 90), causing the oxygen-hemoglobin dissociation curve to be shifted to the left, which may impact tissue ischemia in the face of inadequate erythropoiesis. Clearly, the presence of fetal hemoglobin should be considered in children less than 1–2 months of age who sustain burn injury, as younger infants (<6 months) thus have lower oxygen carrying capacity. This is exacerbated by a decreased production of erythropoietin in response to hypoxia or anemia in critically ill infants with sepsis or polytrauma [8]. Burned children clearly fall into this category. Children who sustain severe burns at birth or shortly after birth due to bathing rituals are at particular risk.

### Metabolic considerations in pediatric blood transfusion

The higher blood transfusion/unit volume ratio in children increases their risk for metabolic perturbation with blood transfusion. Both the red blood cells themselves and the substances used to help preserve red blood cells contribute to these effects. Risks related to transfusion include hyperkalemia, hypomagnesemia, hypothermia, acidosis, and hypothermia.

Hyperkalemia associated with blood transfusion poses a significant risk in children, and potassium levels should be monitored in children receiving >20 ml/kg transfusion volume (or lower if the patient has renal dysfunction or hyperkalemia at the onset of transfusion). Hyperkalemia has been associated with cardiac arrest during large blood volume transfusions intraoperatively in children and infants receiving exchange transfusions [9, 10]. Children with small blood volumes are at particularly high risk of hyperkalemia due to both volume/size considerations and the developing renal function of infants and small children. Potassium levels differ among blood products. Whole blood, irradiated units, and units nearing the expiration date (i.e., “old blood”) contain the largest amounts of potassium [11, 12]. Practices that decrease hyperkalemic cardiac arrest risk include using “young” blood (packed red blood cell (PRBC) <7 days in age), washing erythrocytes prior to transfusion, and avoiding whole blood transfusion in small infants. The life-threatening arrhythmias associated with rapid large volume can be ameliorated by administering calcium [9, 12]. Administration of calcium treats hyperkalemic arrhythmias by opposing the effects of hyperkalemia on the heart’s electrical conduction system. Additional measures, such as intravenous glucose, insulin, albuterol, and Kayexelate, may be needed to resolve the hyperkalemia.

In addition to ameliorating hyperkalemia, ionized calcium is an important cofactor in infant coagulation and myocardial contractility [13]. Citrate, used in blood storage to prevent clotting, prevents clot formation by chelating calcium. As such, transfusion may induce hypocalcemia. The type of blood product transfused, the rate of the transfusion, and the patient hepatic function all influence the extent of hypocalcemia [5, 14]. Whole blood and fresh frozen plasma (FFP) contain the highest concentration of citrate/unit volume of product; hence, they have the highest hypocalcemia risk. Hypocalcemia has been reported after the transfusion of FFP [15]. The neonate is at a particular risk of cardiac dysfunction with hypocalcemia due to the relative lack of neonatal cardiac sarcoplasmic reticulum. This reduction makes the neonate myocardium dependent on ionized calcium for both normal contraction and relaxation. Transfusing blood at a rate of less than 1 ml/kg/min may ameliorate the hypocalcemic effect of the blood. Correction of hypocalcemia can be accomplished with administration of either calcium chloride (5–10 mg/kg) or calcium gluconate (15–30 mg/kg) intravenously. In general, the dose of calcium gluconate required to achieve the same effect is three times that of calcium chloride. Because calcium may result in clot formation when in contact with blood, calcium should never be administered in a blood line. Magnesium, which is often altered in association with calcium, must also be considered. Hypomagnesemia may also occur after massive transfusion, and if a patient is hypocalcemic, magnesium levels should be obtained. Magnesium stabilizes resting membrane potential; hence, hypomagnesemia may cause life-threatening arrhythmias. If ventricular fibrillation or ventricular tachycardia develops after transfusion and does not respond to calcium administration, intravenous magnesium sulfate, in a dose of 25–50 mg/kg, may be helpful.

Environmental issues also impact transfusion effects. Hypothermia in burned children, in particular, requires special consideration. Children, due to their large surface area to volume ratio, are at increased risk for hypothermia. Not only do children with burn injury lose skin integrity, and hence the key temperature regulating mechanism, they also actively lose heat through convection and conduction through wet wounds and exposed tissue. Hypothermia will increase oxygen consumption and exacerbate coagulopathy and is associated with increased mortality [16, 17]. Hypothermia may be exacerbated during periods of rapid transfusion utilizing cold blood products, especially during episodes of massive transfusion in the operating theater. Hypothermia may be ameliorated using several different methods, including the use of blood warmers during transfusion, increased ambient room temperature, external warming devices, and potentially warming central venous catheters.

Hypothermia frequently accompanies another significant complication of transfusion in children: acidosis. Hypovolemia in the operating room during massive excision is of particular concern with respect to the development of acidosis. As such, life-threatening acidosis may occur during rapid transfusion for massive blood loss in a hypovolemic patient. Because stored blood cells continue to metabolize, lactic acid increases in stored blood, making acidosis more likely. It is also notable that metabolic alkalosis may occur several days after massive transfusion from the metabolism of the citrate in the blood products administered.

### Infectious disease transmission

Although transmission of infectious diseases due to blood transfusion has decreased over time, the transmission of infectious diseases remains an important problem in children requiring blood transfusion [2]. Parents, understandably, are worried about hepatitis and human immunodeficiency virus from blood transfusion. Blood products in different countries differ in the frequency of transmission of infectious organisms. Current blood screening tests include hepatitis B surface and core antigen, hepatitis C virus antibody, HIV-1 and HIV-2 antibody, HTLV-I and HTLV-II antibody, nucleic acid amplification testing for HIV-1 and HCV, syphilis, and West Nile virus [18]. In addition to these commonly measured viral infections, bacteria can also infect blood products. The incidence of bacterial contamination is highest for platelets [19–21]. Other potential infections that could be transmitted via transfusion that are not tested for include HTLV, West Nile virus, babesiosis, Chagas disease, Lyme disease, malaria, Creutzfeldt-Jakob disease, and severe acute respiratory syndrome (SARS). Screening for Zika and Ebola virus has recently been released by the Food and Drug Administration [22].

### Incompatibility/immunologic factors

Hemolytic transfusion reactions continue to occur despite the careful application of compatibility testing. Blood mismatch transfusions is due primarily to clerical error. Particularly important is the verification of blood products prior to transfusion by physician and nurse with the patient’s identification to make sure that the unit is truly intended for that patient. This simple, inexpensive procedure can prevent a life-threatening transfusion reaction. Strict adherence to transfusion protocols is important to avoid this iatrogenic complication.

Acute hemolytic reactions generally occur due to ABO incompatibility and causes immunologic destruction of red cells. However, this complication can also occur due to minor antigens not detected by current screening techniques [23, 24]. Anaphylactic reactions rarely occur. Transfusion-related graft-versus-host reaction, in which the lymphocytes in the transfused blood cause host cell destruction, occurs primarily in immunocompromised patients and has been reported in neonates and immunocompromised children [25–28]. This condition occurs primarily in premature infants or children with rapid acute blood loss, cardiopulmonary bypass, cancer, or severe systemic illness [29]. Burned children are immunosuppressed and require massive transfusions in the operating room, thus putting them at risk for this complication. Transfusion-related graft-versus-host disease can be reduced by using irradiated units, which effectively decrease the lymphocyte count. However, since irradiated blood has a higher potassium content than nonradiated blood, potassium levels must be monitored closely.

### Determination of blood transfusion volume in a child with burn injury

The child’s blood volume varies with age and weight; hence, the amount of blood required in times of acute blood loss varies markedly among children of different ages. The highest blood volume per unit weight is for a premature infant (90–100 ml/kg), while the lowest is for a very obese child (65 ml/kg). A term infant has an 80–90 ml/kg blood volume until age of 3 months, after which the total blood volume drops to 70 ml/kg [2]. The difference in total blood volume in an infant compared to that in an adult is an important consideration in determining how much blood to transfuse in a child. As such, formulas have been developed to guide clinicians during massive blood loss (blood loss greater than 1 blood volume) in a child without preexisting anemia. The blood loss at which transfusion should be considered in a child (or an adult) without preexisting anemia (maximal allowable blood loss (MABL)) can be estimated from the following formula [30]:

$$ \mathrm{MABL}=\left[\left({\mathrm{Hct}}_{\mathrm{start}}\hbox{--}\ {\mathrm{Hct}}_{\mathrm{target}}\right)/{\mathrm{Hct}}_{\mathrm{start}}\right] \times \mathrm{E}\mathrm{B}\mathrm{V} $$

Theoretically, blood loss amounting to the MABL can be replenished by crystalloid or colloid, with blood transfusion reserved for higher blood losses. In general, the hematocrit in PRBC approaches 70%; hence, approximately 0.5 ml of packed RBCs should be transfused for each milliliter of blood loss beyond the MABL. Although this formula provides a framework for blood transfusion, it is merely an estimate. Ultimately, blood transfusion requires careful consideration of patient condition, local resources, and severity of illness. A burned child poses a particular challenge, due to the increased red cell destruction and decreased red cell production that accompanies major burn injury. Surgical excision of the burn wound results in major blood loss; a child loses 5% of a blood volume per percent face burn excised and 2% of a blood volume per percent burn excised on other areas [31]. Thus, an infant having burn excision of the entire head could potentially lose 90% of total blood volume (body surface area of the head of 18 percent × 5 percent blood volume lost per percent excision of the head). Sufficient units of blood products should be ready prior to the onset of surgery.

The optimal transfusion threshold for critically ill children has been evaluated in a multicenter trial in pediatric intensive care units [32]. This study reported that a restrictive transfusion strategy, which transfused at a hemoglobin <7 g/dL, had mortality and outcomes comparable to a liberal strategy (maintain hemoglobin >10 g/dL). This study evaluated stable, critically ill children without acute blood loss; hence, its applicability to burn patients is limited. A recently completed randomized prospective trial in adult burn patients with burn size >20% TBSA demonstrated no outcome difference between different transfusion strategies (Palmieri, in press).

Massive blood transfusion may result in the lethal triad: hypothermia, acidosis, and coagulopathy. Hypothermia in the operating room, as discussed above, is more prevalent in the infant due to the larger surface area per unit mass. Hypothermia is further exacerbated by exposure to the cold operating room suite and anesthetic agents which decrease shivering. Acidosis due to hypovolemia and hypothermia develops if patients are under-resuscitated. Coagulopathy, the final link in the triad, occurs during massive blood transfusion as a result of depletion of clotting factors. Currently, PRBCs are the predominant form of red cell transfusion. Since 80% of coagulation factors are separated from PRBCs during processing, clotting factor deficiency generally occurs at approximately 1 blood volume [33]. However, if whole blood is used, all clotting factors except for labile factors V and VIII will be transfused at normal levels. Thus, coagulation abnormalities tend to occur later (>3 blood volumes) when using whole blood [34]. However, whole blood carries substantial risks as well, including hyperkalemia, transfusion reactions, and transfusion-related circulatory overload.

Thrombocytopenia may be caused by dilution of platelets during transfusion. In general, a patient will lose 40% of the starting platelet count in the first blood volume loss, with loss of an additional 20% of the starting count at a second blood volume [33]. It is thus important to record the platelet count prior to an anticipated massive blood loss, such as happens with major burn excision. A child with sepsis and a low starting platelet count is far more likely to require platelet transfusion than a child with a high or normal platelet count. The optimal ratio of fresh frozen plasma to packed red blood cells in massive bleeding associated with extensive surgical burn excision has not been definitively defined; however, a prospective trial in burned children suggests that a 1:1 FFP/PRBC strategy may improve outcomes.

### Complications of blood transfusion

The use of PRBC and other transfusion products also predisposes patients to other potential complications, including transfusion-related immunomodulation (TRIM), transfusion-related acute lung injury (TRALI), and transfusion-related circulatory overload (TACO). As blood is stored, it releases a variety of agents, including toxic oxygen radicals, cytokines, soluble HLA class I antigens, histamine, plasminogen activator inhibitor-1, and leukocyte elastase [35]. Older blood may increase infection risk in multiple different patient populations [36]. Blood transfusion in general impact the immune system by increasing suppressor T lymphocyte and natural killer cell function, depressing monocyte and macrophage phagocytic activity, inducing immune cell anergy and clonal deletion, decreasing macrophage antigen presentation, suppressing lymphocyte blastogenesis, decreasing delayed-type hypersensitivity, and suppressing mitogen-stimulated human T cell proliferation [37]. TRIM involves both immune activation (such as transfusion reactions, TRALI, alloimmunization, autoimmune diseases, and transfusion-associated graft-versus-host disease) as well as immune tolerance and immunosuppression (infection, cancer recurrence, microchimerism, enhanced allograft survival). TRALI, first described in 1983, is characterized by respiratory distress, hypoxemia, pulmonary edema, hypotension, and fever after receiving blood transfusion. A recent study in Canada estimated that the incidence of TRALI in children is 1.8/100,000 population, much less than in adults [38]. The incidence of TRALI in burn injury is unknown. TACO consists of pulmonary edema that develops within 6 h of transfusion due to increases in hydrostatic pressure. The incidence of TACO is <11% in adults and carries a 5–15% mortality [39]. The incidence of TACO in burned children and adults has not been determined.

Multiple strategies can be employed to decrease the immunologic and storage-related impact of blood. The first strategy is to decrease the amount of blood lost due to testing and surgery. For example, reducing volume and frequency of blood draws, adopting a restrictive transfusion policy, and utilizing tourniquets and tumescence during surgical burn excision will all decrease the volume of blood removed from the patient. The second strategy is to minimize the volume of blood administered. This entails using leukoreduced blood, transfusing blood one unit at a time, and investigating alternatives to transfusion. The fewer units of blood the patient receives, the less likely the patient is to have a transfusion-related complication.

## Conclusions

Children, due to their age-dependent physiology, alterations in body mass ratio, and immature cardiac and immunological status, have variable and complex transfusion needs after burn injury. Optimizing the treatment of burn-injured children requires knowledge of these issues and careful consideration of the impact of transfusion on patient outcomes. Fastidious attention to the sometimes subtle differences between children and adults is needed to optimize blood utilization in children with major burn injury.

## Abbreviations

EBV: Estimated blood volume
FFP: Fresh frozen plasma
Hct: Hematocrit
HCV: Hepatitis C virus
HIV: Human immunodeficiency virus
MABL: Maximal allowable blood loss
PRBCs: Packed red blood cells
SARS: Severe acute respiratory syndrome
TACO: Transfusion-related acute circulatory overload
TBSA: Total body surface area
TRALI: Transfusion-related acute lung injury
TRIM: Transfusion-related immunomodulation
